# Fecal Bacterial Community and Metagenome Function in Asians with Type 2 Diabetes, According to Enterotypes

**DOI:** 10.3390/biomedicines10112998

**Published:** 2022-11-21

**Authors:** Xuangao Wu, Sunmin Park

**Affiliations:** 1 Department of Bioconvergence, Hoseo University, Asan 31499, Republic of Korea; 2 Department of Food and Nutrition, Institute of Basic Science, Hoseo University, Asan 31499, Republic of Korea

**Keywords:** enterotypes, machine learning approach, *Bifidobacterium*, *Faecalibacterium prausnitzii*, parasympathetic nervous system, gut dysbiosis

## Abstract

The role of gut microbes has been suggested in type 2 diabetes (T2DM) risk. However, their results remain controversial. We hypothesized that Asians with T2DM had different fecal bacterial compositions, co-abundance networks, and metagenome functions compared to healthy individuals, according to enterotypes. This hypothesis was examined using the combined gut microbiota data from human fecal samples from previous studies. The human fecal bacterial FASTA/Q files from 36 different T2DM studies in Asians were combined (healthy, *n* = 3378; T2DM, *n* = 551), and operational taxonomic units (OTUs) and their counts were obtained using qiime2 tools. In the machine learning approaches, fecal bacteria rich in T2DM were found. They were separated into two enterotypes, Lachnospiraceae (ET-L) and Prevotellaceae (ET-P). The Shannon and Chao1 indices, representing α-diversity, were significantly lower in the T2DM group compared to the healthy group in ET-L (*p* < 0.05) but not in ET-P. In the Shapley additive explanations analysis of ET-L, *Escherichia fergusonii*, *Collinsella aerofaciens*, *Streptococcus vestibularis*, and *Bifidobacterium longum* were higher (*p* < 0.001), while *Phocaeicola vulgatus*, *Bacteroides uniformis*, and *Faecalibacterium prausnitzii* were lower in the T2DM group than in the healthy group (*p* < 0.00005). In ET-P, *Escherichia fergusonii*, *Megasphaera elsdenii*, and *Oscillibacter valericigenes* were higher, and *Bacteroides koreensis* and *Faecalibacterium prausnitzii* were lower in the T2DM group than in the healthy group. In ET-L and ET-P, bacteria in the healthy and T2DM groups positively interacted with each other within each group (*p* < 0.0001) but negatively interacted between the T2DM and healthy groups in the network analysis (*p* < 0.0001). In the metagenome functions of the fecal bacteria, the gluconeogenesis, glycolysis, and amino acid metabolism pathways were higher, whereas insulin signaling and adenosine 5′ monophosphate-activated protein kinase (AMPK) signaling pathways were lower in the T2DM group than in the healthy group for both enterotypes (*p* < 0.00005). In conclusion, Asians with T2DM exhibited gut dysbiosis, potentially linked to intestinal permeability and the enteric vagus nervous system.

## 1. Introduction

Type 2 diabetes (T2DM) has seen a remarkable rise in Asians over the last two to three decades. Several differences have been observed between Asians and Caucasians in their innate biological susceptibilities and risk factors for the development of T2DM [1]. Unlike Caucasians, Asians with T2DM are non-obese and have normoinsulinemia [1]. Asians have lower insulin secretion capacities and β-cell masses with smaller islets than Caucasians and are thus more susceptible to T2DM [1]. T2DM incidence rises when insulin resistance increases [1]. Since Asians have traditionally consumed a low-fat diet with multigrain rice and vegetables, they have been non-obese and insulin-sensitive in the past [2]. They were thus at low risk of T2DM. However, the westernization of lifestyles, including a high-fat diet and low physical activity, has increased insulin resistance in recent years [3], which is related to the recent increase in T2DM incidence in Asians [4].

Dietary patterns are known to be associated with the gut microbiota, which interacts with the host’s metabolism [5]. The bacteria inhabit the gastrointestinal tract, mainly the colon [5]. Gut microbiota communities are altered with varying diets, and the host’s digestion capacity, including the secretion and reabsorption of gastric acid, pancreatic juices, bile acid, and intestinal permeability, is also changed [6]. These changes influence glucose metabolism in the host, contributing to the development and exacerbation of T2DM. About 100 trillion microbes colonize the gut, predominantly in the colon, but most have not yet been cultured or had their characteristics identified. The gut microbiota is a complex ecosystem that influences the host’s metabolism. Gut microbes regulate energy, glucose, amino acid, and fatty acid metabolism as well as immunity, oxidative stress, short-chain fatty acid (SCFA) production, endocrine-disrupting chemical elimination, and hormone action, thereby modulating the host’s metabolism [7]. Gut microbial dysbiosis is an alteration of the diversity and abundance of intestinal microbes that can lead to several disorders. An increase in opportunistic pathogens and a decrease in beneficial bacteria in dysbiosis influences the pathologies of not only immune diseases but also various metabolic diseases, including obesity and T2DM [8,9]. Dysbiosis disrupts the gut’s immune homeostasis and can lead to endotoxemia, chronic inflammation, and intestinal permeability [10]. Even though the exact gut-microbiota-related T2DM pathophysiology remains unclear, preliminary reports suggest that manipulating the gut microbiota community may prevent and alleviate T2DM.

Due to the enormous number of gut microbes, their heterogenous composition, and their network, bacteria to induce metabolic diseases are difficult to isolate and study. Instead, the gut microbiota has been grouped into several clusters called enterotypes, which mainly include Bacteroides (ET-B), Ruminococcus (ET-R), and Prevotella (ET-P) [11]. These clusters are formed due to the long-term diet, the host’s genetic properties, and intestinal permeability (a sign of a perturbed barrier function) [12]. The parasympathetic nervous system (PNS) also influences the networks and clusters of gut microbiota [13]. Predominantly, the Bacteroides enterotype (ET-B) is associated with high protein and animal fat diets, Prevotella (ET-P) is associated with carbohydrate-rich diets, and Ruminococcus (ET-R) is associated with eating a balanced diet [14]. The enterotype is also linked to host digestion and absorption and is not easily altered. However, sustained dramatic dietary changes, including the consumption of prebiotics, can modulate the increment or decrement of gut microbes in the co-occurrence network [15,16]. When specific opportunistic pathogens increase in the network, the gut bacteria induce dysbiosis, which is directly related to disease induction and exacerbation. This gut dysbiosis in a specific disease is called a disease enterotype, which is dominantly composed of *Escherichia* and *Clostridium* in intestinal diseases such as colon cancer, Crohn’s disease, and irritable bowel disease [17]. Specific prebiotics can alter the co-occurrence of gut microbes, thus decreasing opportunistic microbes and preventing disease pathogenesis [15]. 

Although patients with T2DM have not been shown to harbor specific gut bacteria assigned as a disease enterotype, T2DM is known to alter gut microbes. Several studies have shown bacterial changes at the genus level, with a predominance of Bifidobacterium, Bacteroides, Roseburia, Faecalibacterium, and Akkermansia being inversely correlated with T2DM [10,18,19]. The association, however, remains controversial. Most studies have included a small number of participants, and a network analysis of the bacteria, which could show the interaction of bacteria to modulate T2DM risk according to enterotype, has not been conducted. In the present study, we hypothesized that Asians with T2DM had a different fecal bacterial composition with their network and metagenome functions compared to healthy Asians, according to enterotypes. This hypothesis was examined using the combined gut microbiota data from human fecal samples from earlier studies. This study showed that fecal bacteria are related to T2DM and that potential therapies to prevent and attenuate its risk could be suggested. 

## 2. Methods

### 2.1. Collection of Fecal Bacteria FASTA/Q Files for T2DM and Healthy Asians

**Figure 1:**
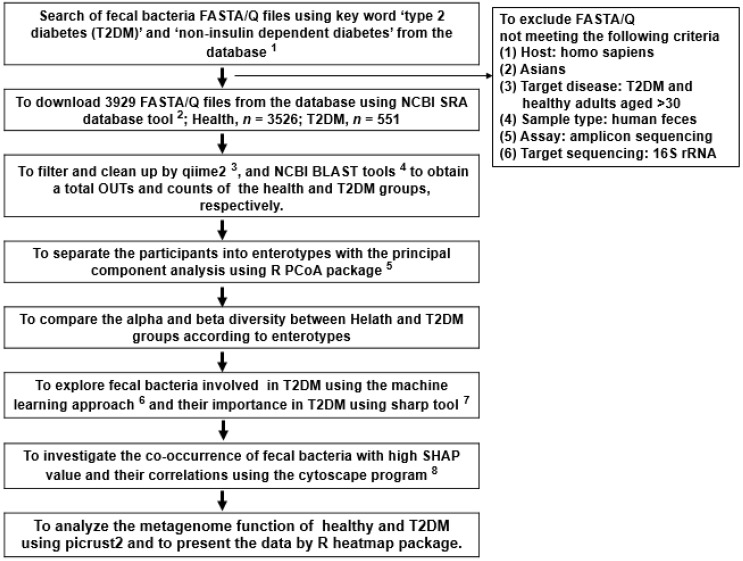
A flow chart of the overall fecal FASTA/Q selection process and analysis methods. ^1^ SRA accession list on NCBI SRA database (https://www.ncbi.nlm.nih.gov/sra, accessed on 14 January 2022) and GMrepo database (https://gmrepo.humangut.info/ accessed on 10 January 2022); ^2^ https://trace.ncbi.nlm.nih.gov/Traces/sra/sra.cgi?view=software, accessed on 10 January 2022; ^3^ https://view.qiime2.org/, accessed on 28 February 2022); ^4^ https://blast.ncbi.nlm.nih.gov/Blast.cgi (accessed on 10 March 2022); ^5^ https://cran.r-project.org/web/packages/aPCoA/index.html, accessed on 21 March 2022; ^6^ https://xgboost.readthedocs.io/en/stable/install.html accessed on 4 April 2022; ^7^ https://shap.readthedocs.io/en/latest/index.html, accessed on 10 January 2022; ^8^ https://cytoscape.org, accessed on 20 April 2022.

A flow chart of the overall fecal FASTA/Q selection and analysis procedures is presented in Figure 1. The FASTA/Q files of fecal bacteria from projects to study the fecal bacterial composition of T2DM and healthy adults were collected from the National Center for Biotechnology Information (NCBI) database (https://www.ncbi.nlm.nih.gov/ accessed on 14 January 2022), the European Nucleotide Archive (ENA) browser (www.ebi.ac.uk/ena/browser/home, accessed on 10 February 2022), and the data repository for Gut Microbiota (GMrepo database; https://gmrepo.humangut.info/, accessed on 10 January 2022) until April 2022. The FASTA/Q files were selected based on the following inclusion criteria: (1) host: homo sapiens, (2) Asians, (3) target participants: T2DM and healthy adults aged over 30 years, (4) sample type: human feces, (5) assay: amplicon sequencing (Miseq), and (6) target sequencing: 16S rRNA. Each study had ethical approval from the corresponding institutional review board from the respective institute, and participating patients volunteered to provide fecal samples and signed informed consent. The 3929 fecal FASTA/Q files, including 3378 healthy and 551 T2DM participants, were collected from studies conducted in China, India, Japan, and Thailand (Table 1). Only some participants provided age and gender: their average age was about 44 years, and the genders were almost equally distributed. 

### 2.2. Fecal Bacterial Composition and Community Analysis 

The fecal FASTA/Q files from humans were downloaded to meet the inclusion criteria and were extracted using the National Center for Biotechnology Information (NCBI) Sequence Read Archive (SRA) toolkits (https://trace.ncbi.nlm.nih.gov/Traces/sra/sra.cgi?view=software, accessed on 23 February 2022; National Center for Biotechnology Information, Bethesda, MD, USA). Using qiime2 (https://view.qiime2.org/, accessed on 28 February 2022), the double-ended sequences of the collected FASTA/Q files were merged using the “make.contigs” command. The sequences were aligned with the SILVA v 1381 database, and nontarget sequences such as mitochondria, archaea, fungi, and unknown sequences were eliminated. The fecal bacterial sequences were preclustered, and chimeras were removed using the “chimera.vsearch” command. The sequences were then clustered with 97% similarity. The taxonomy of the operational taxonomic units (OTUs) was annotated according to NCBI Basic Local Alignment Search Tool (BLAST) (https://blast.ncbi.nlm.nih.gov/Blast.cgi, accessed on 10 March 2022; National Center for Biotechnology Information, Bethesda, MD, USA)). The 2647 representative sequences were obtained, and their biome files containing taxonomy and counts were used for further analysis. 

### 2.3. Enterotypes

Using the taxonomy and counts of fecal bacteria from all the collected fecal FASTA/Q files, the enterotypes were separated by principal component analysis (PCA). The number of enterotypes was assigned to satisfy eigenvalues > 1.5 using the “FactoMineR” and “Factoextra” packages using R software [14]. The optimal number of clusters was 2. Two enterotypes were assigned and named with the predominant bacteria at the family level. The main bacteria in enterotypes 1 and 2 were Lachnospiraceae (ET-L) and Prevotellaceae (ET-P), respectively. ET-L and ET-P included 3515 (healthy: 3043; T2DM: 472) and 414 (healthy: 335; T2DM: 79) participants, respectively. The predominant fecal bacteria for T2DM risk were determined in ET-L and ET-P. 

### 2.4. α-Diversity, β-Diversity, and Linear Discriminant Analysis (LDA) Scores 

Many bacteria exist in the gut, and their types and diversity play critical roles in host metabolism and health status. Alpha-diversity (α-diversity) is the species diversity in a person’s gut, and it was represented with the Chao1, Shannon, and Ace indices. Its metric was calculated with a “summary.single” command in the Mothur software package (https://mothur.org/, accessed on 21 March 2022). Beta-diversity (β-diversity) is the ratio between regional and local species diversity that demonstrates the separation between the groups [20]. The β-diversity of the bacteria was estimated with the clearcut command in Mothur to construct a phylogenetic tree. The “unifrac.unweighted” command was applied to calculate the unweighted UniFrac distance matrix, and a principal coordinate analysis (PCoA) was used for visualization. The PCoA clustered the FASTA/Q samples into the healthy and T2DM groups with good separation. The statistical difference between the healthy and T2DM groups was checked with a permutational multivariate analysis of variance (PERMANOVA) test. LDA scores represent the effect size of each abundant species, and they were analyzed with the lefse command in the Mothur program.

### 2.5. XGBoost Classifier Training and Shapley Additive Explanations (SHAP) Interpreter

According to the enterotype, the specific predominant fecal bacteria in T2DM were analyzed with a machine learning approach, including XGBoost, random forest, and linear regression, and compared with the healthy group. The fecal data were divided randomly into 80% for the training set and 20% for the testing set. The best hyperparameter settings were found using a random grid search with 1000 iterations of the XGBoost algorithm using the Scikit package [21]. The best model for explaining the healthy and T2DM groups was generated. The receiver operating characteristic (ROC) area for the best model was determined using the training and test sets of the fecal bacteria. The 10-fold cross-validation was calculated using the cross_val_score function in the test data set. For its calculation, the original training data were clustered into ten subsets: nine sets were used as training data, and one set was used as test data and iterated ten times [22]. The accuracy, specificity, and sensitivity were generated from ten data sets. The 0.9 value of the 10-fold cross-validation indicated that the accuracy of the selected model was 90% [23]. 

The SHAP analysis separated the bacteria positively involved in the T2DM and healthy groups using the output of the XGBoost model [23,24]. It provided the SHAP value of each species of bacteria to the classifier using the SHAP (0.39.0) package. 

The correlation of the bacterial species was conducted using a Pearson correlation analysis, and a network analysis was carried out using the correlation results to determine the gut microbes using the Cytoscape program downloaded from the website https://cytoscape.org/accessed on 20 April 2022; U.S. National Institute of General Medical Sciences, Bethesda, MD, USA). 

### 2.6. Metagenome Function of Fecal Bacteria by Picrust2

The metagenome functions of the fecal bacteria were estimated from the genes they contained using the FASTA/Q files. These functions were determined with the Phylogenetic Investigation of Communities by Reconstruction of Unobserved States (Picrust2) software, which is used for predicting functional abundances based only on marker gene sequences [25]. The metabolic functions of the genes in the fecal bacteria were estimated based on the Kyoto Encyclopedia of Genes and Genomes (KEGG) orthologues (KO) and mapped using the KEGG mapper (https://www.genome.jp/kegg/tool/map_pathway1.html; accessed on 18 May 2022; Kyoto University, Kyoto, Japan) [25]. 

### 2.7. Statistical Analysis

The statistical analysis was performed using SAS version 7 (SAS Institute; Cary, NC, USA) and the R package. The data are expressed as the means ± standard deviations (SD), and the statistical significance was set at *p* < 0.05. The mean statistical differences between the T2DM and healthy groups were determined using a two-sample t-test and enterotypes. The visualization of the data was conducted using R-studio and the ggplot2 package. 

## 3. Results

### 3.1. Enterotypes of Fecal Bacteria

**Figure 2:**
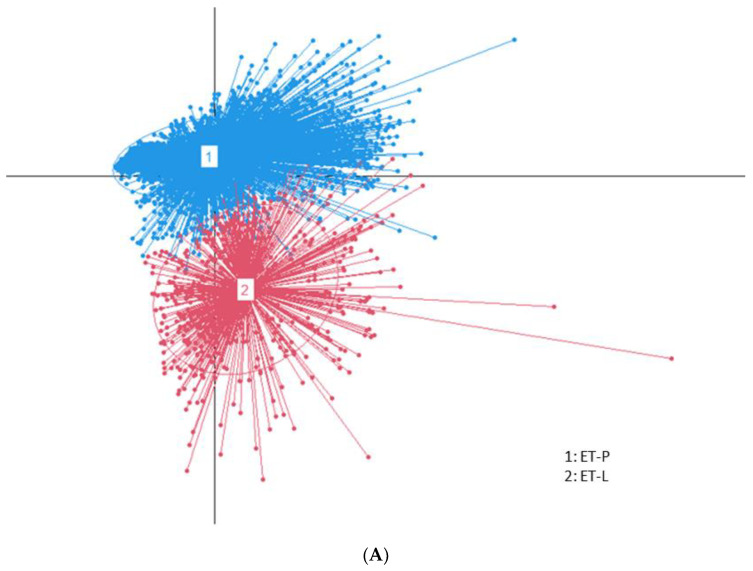

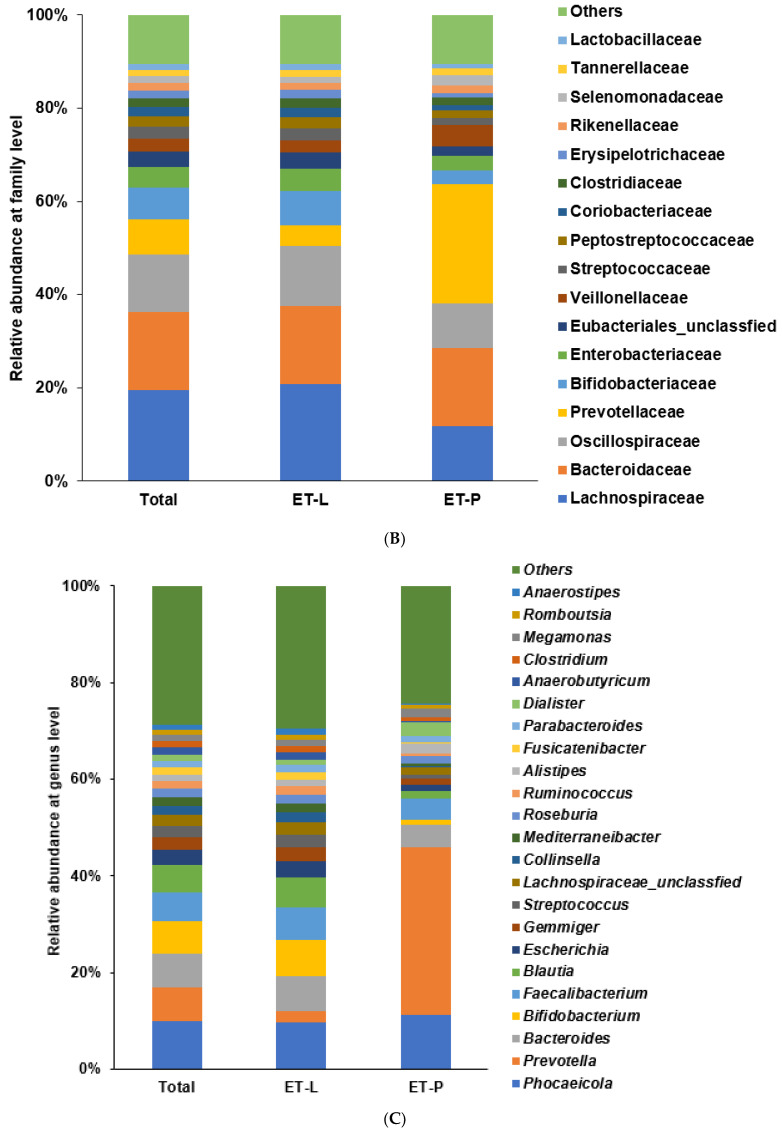
Fecal bacteria stratification into two enterotypes and bacterial composition in each enterotype. (**A**) Fecal bacteria clustering (two enterotypes). Red and blue colors indicated ET-L and ET-P communities, respectively; (**B**) Relative abundance of fecal bacteria in each enterotype at the family level; (**C**) Relative abundance of fecal bacteria in each enterotype at the genus level; ET-L: Enterotype Lachnospiraceae; ET-P: Enterotype Prevotellaceae.

All species (*n* = 2647) of fecal bacteria were clustered into two enterotypes according to meeting eigenvalue > 1.5 in the PCA analysis (Figure 2A). The separated enterotypes were assigned to Lachnospiraceae (ET-L) and Prevotellaceae (ET-P) according to the fecal bacteria in each cluster. ET-L was mainly composed of Lachnospiraceae, Oscillospiraceae, and Bifidobacteriaceae. In contrast, ET-P was rich in Prevotellaceae (Figure 2B). At the genus level, *Bacteroides*, *Bifidobacterium*, *Faecalibacterium*, *Blautia*, *Germiger*, and *Escherichia* were relatively abundant in ET-L, and *Prevotella* was the primary species in ET-P. The fecal bacteria related to T2DM were found in ET-L and ET-P (Figure 2C). 

### 3.2. α- and β-Diversity of Fecal Diversity 

The Chao1 (*p* = 0.045) and Shannon indices (*p* = 0.046), representing α-diversity, were lower in the T2DM compared to the healthy group in ET-L (*p* < 0.05; Supplementary Figure S1A,B). In ET-P, the Chao1 (*p* = 0.917) and Shannon indices (*p* = 0.264) showed no variation between the healthy and T2DM groups (Supplementary Figure S1C,D). 

In ET-L and ET-P, the β-diversity measured by the Bray–Curtis dissimilarity matrix was significantly different between the healthy and T2DM groups (*p* < 0.001), indicating that the fecal bacterial composition was different between the healthy and T2DM groups (Supplementary Figure S2A,B). 

### 3.3. Fecal Bacterial Composition of T2DM Patients in Each Enterotype 

At the phylum level, Firmicutes and Bacteroidetes were lower and Actinobacteria and Verrucomicrobia were higher in the T2DM group compared to the healthy group in ET-L. However, the ratio of Firmicutes/Bacteroidetes (F/B) was higher in the T2DM group (2.88 ± 0.32) than in the healthy group (38 ± 0.08; *p* < 0.05) in ET-L (Supplementary Figure S3A,B). The relative abundance of Firmicutes and Bacteroidetes in ET-P was the opposite of what was seen in ET-L; the relative abundance of Firmicutes was higher in T2DM than in the healthy group. However, the F/B ratio was higher in the T2DM group (0.88 ± 0.05) than in the healthy group (58 ± 0.02; *p* < 0.001; Supplementary Figure S3C). Interestingly, the level of Proteobacteria was much higher in the T2DM group than in the healthy group in ET-P but not in ET-L. These results suggested that the fecal bacteria should be studied according to the enterotypes. 

**Figure 3:**
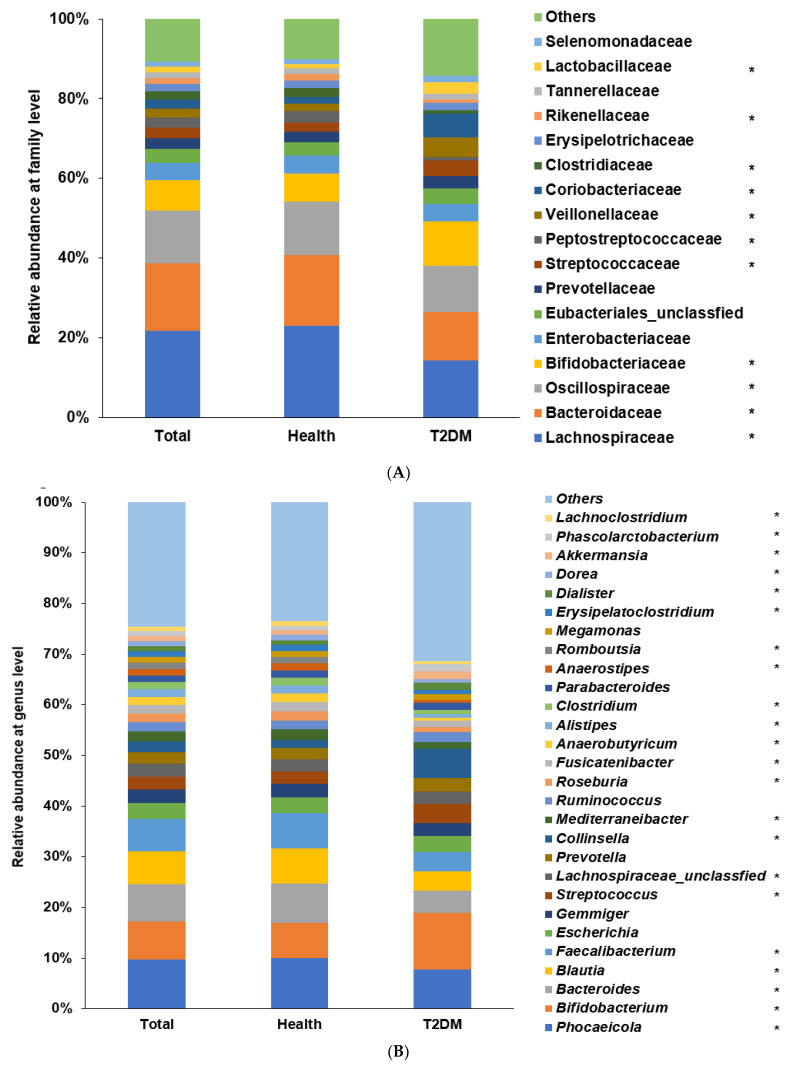

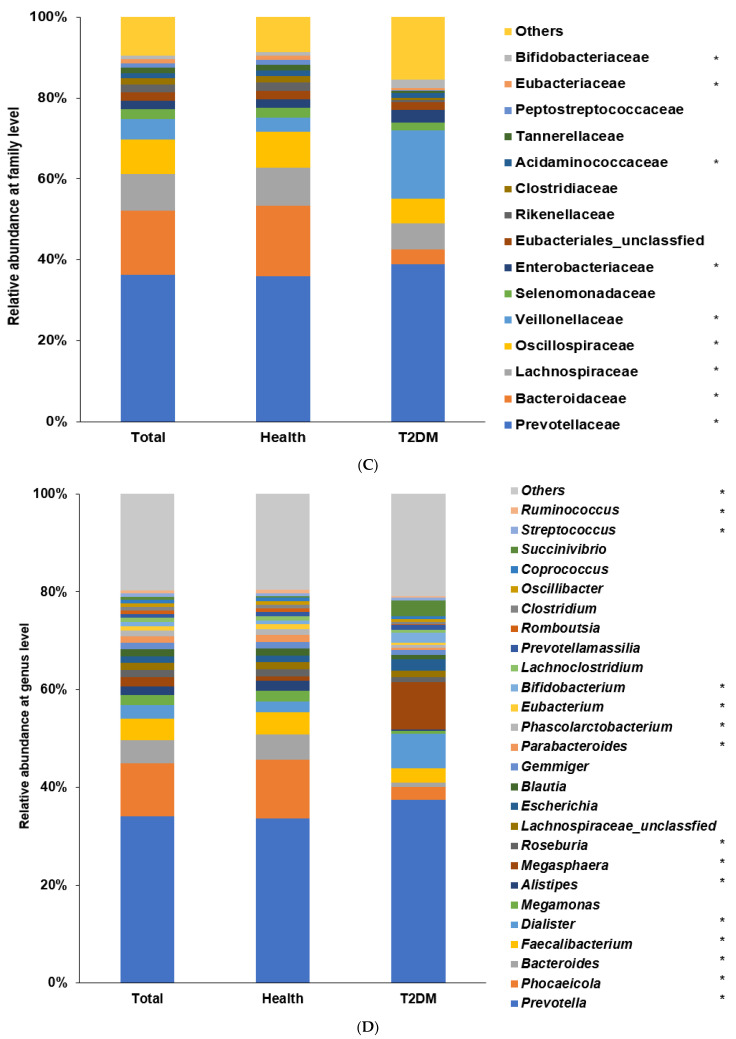
Fecal bacterial compositions between type 2 diabetes (T2DM) and healthy Asians. (**A**) Relative abundance of fecal bacteria in T2DM and healthy adults in ET-L at the family level; (**B**) Relative abundance of fecal bacteria in T2DM and healthy adults in ET-L at the genus level; (**C**) Relative abundance of fecal bacteria in T2DM and healthy adults in ET-P at the family level; (**D**) Relative abundance of fecal bacteria in T2DM and healthy adults in ET-P at the genus level; * Significant differences between T2DM and healthy groups at *p* < 0.00001 (Bonferroni-corrected *p* value). ET-L: Enterotype Lachnospiraceae; ET-P: Enterotype Prevotellaceae.

At the family level, the relative abundance of Lachnospiraceae, Bacteroidaceae, Oscillospiraceae, and Clostridiaceae was higher, while that of Streptococcaceae, Veillonellaceae, Coriobacteriaceae, and Lachnospiraceae was lower in the healthy group than in the T2DM group in the participants with ET-L (*p* < 0.0001; Figure 3A). At the genus level, the relative abundance of *Bacteroides*, *Blautia*, *Dorea*, *Phocaeicola*, *Faecalibacterium*, and *Alistipes* was higher, and that of *Bifidobacterium*, *Collinsella*, *Streptococcus*, and *Dialister* was lower in the healthy group than the T2DM group in the participants with ET-L (*p* < 0.00001; Figure 3B).

At the family level, the relative abundance of Prevotellaceae and Veillonellaceae was lower, but that of Bacteroidaece, Lachinosphiraceae, and Oscillospiraceae was higher in the healthy group than in the T2DM group of participants with ET-P (*p* < 0.0001; Figure 3C). At the genus level, the relative abundance of *Prevotella*, *Dialister*, *Megasphara*, *Escherichia*, *Bifidobacterium*, and *Streptococcus* was higher, but that of *Phocaeicola*, *Bacteroides*, *Faecalibacterium*, *Alistipes*, and *Blautia* was lower in the T2DM group than in the healthy group of the participants with ET-P (*p* < 0.0001; Figure 3D). 

### 3.4. In ET-L, the Prediction of Gut Microbiota Specific to T2DM by the Machine Learning Approach 

**Figure 4:**
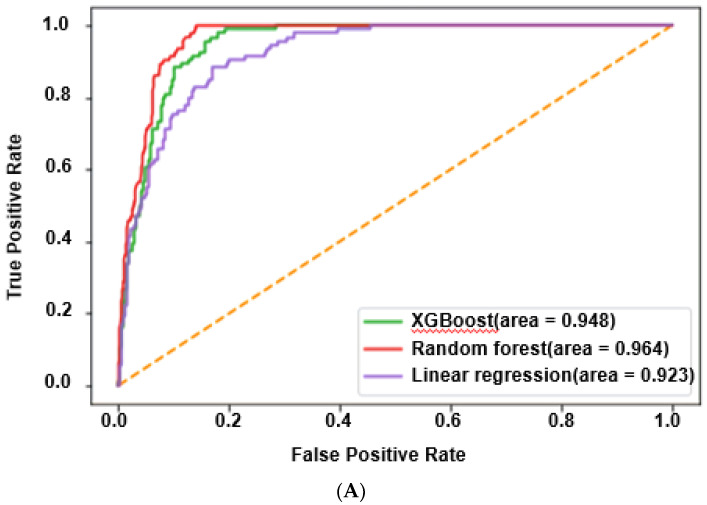

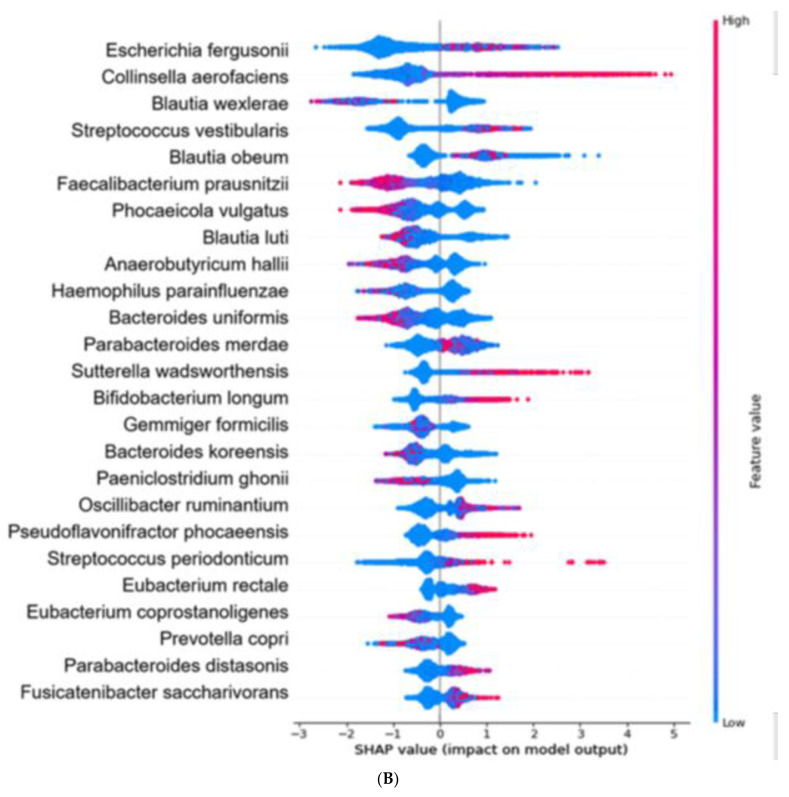

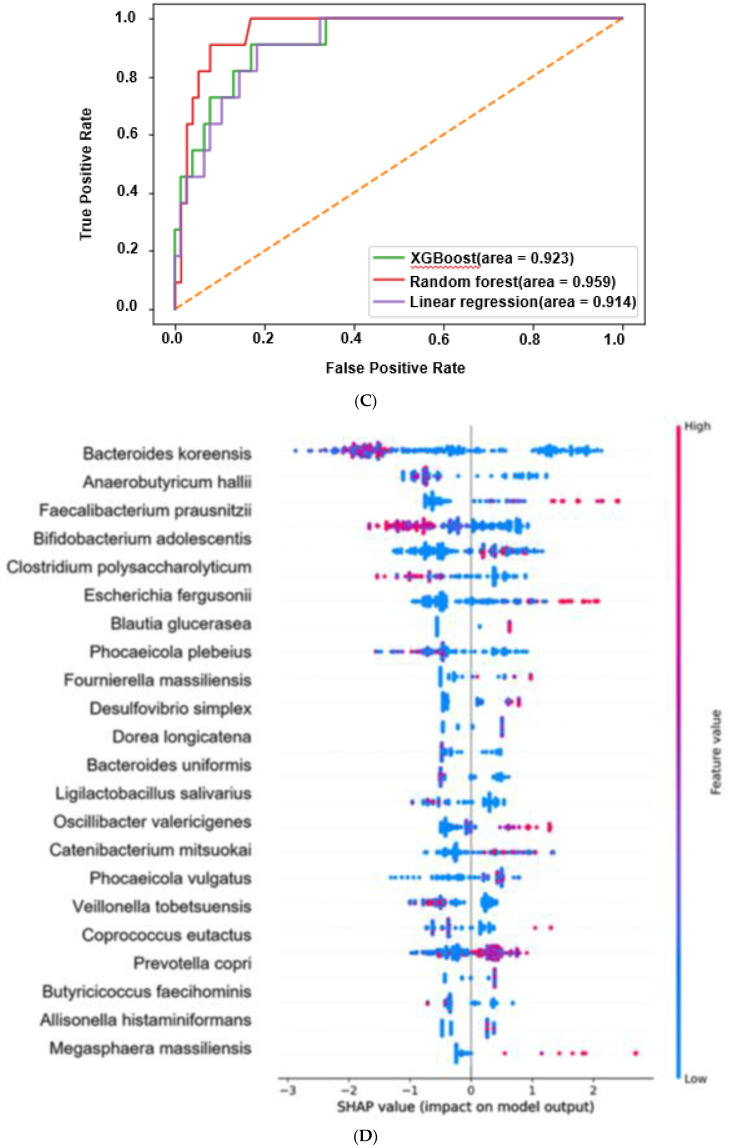
Primary bacteria between the type 2 diabetes (T2DM) and healthy Asians at the species level by XGBoost in each enterotype. (**A**) The area under the curve (AUC) of the receiver operating characteristic (ROC) in the prediction model for fecal bacteria at the species level in the comparison between the T2DM and healthy groups in ET-L; (**B**) Relative abundance of fecal bacteria at the species level between the T2DM and healthy groups in the prediction model in ET-L; (**C**) Area under the curve (AUC) of ROC in the prediction model for fecal bacteria at the species level in the comparison between the T2DM and healthy groups in ET-P; (**D**) Relative abundance of fecal bacteria at the species level between the T2DM and healthy groups in the prediction model in ET-P; ET-L: Enterotype Lachnospiraceae; ET-P: Enterotype Prevotellaceae.

According to the enterotypes, the fecal bacteria related to T2DM risk were predicted using XGBoost, random forest, and linear regression models. In ET-L, 3515 fecal FASTA/Q samples (healthy: *n* = 3043; T2DM: *n* = 472; 13.4% of all participants) were used to generate a prediction model for T2DM risk. The area under the curve (AUC) and 95% CIs for the prediction models are presented in Figure 4A. The AUC of the ROC in the prediction model with 25 species was 0.948, 0.966, and 0.924 in the XGBoost, random forest, and linear regression models, respectively (*p* < 0.0001; Table 2). The results indicated that the prediction models of fecal microbes fit well to explain T2DM risk. The accuracy, sensitivity, specificity, and precision of the prediction model were about 0.90, 0.75, 0.97, and 0.88, and these values indicated that the model was acceptable (*p* < 0.0001; Table 2). 

Among the three models, the model generated by XGBoost was used to identify the species that positively or negatively influenced T2DM risk. The SHAP analysis demonstrated that in ET-L *Escherichia fergusonii*, *Streptococcus vestibularis*, *Collinsella aerofaciens*, *Bifidobacterium longum*, *Oscillibacter valericigenes*, *Lawsonibacter asaccharolyticus*, and *Erysipelatoclostridium ramosum* were positively associated with T2DM risk, while *Phocaeicola vulgatus*, *Bacteroides uniformis*, *Faecalibacterium prausnitzii*, *Blautia luti*, *Anerobutyricum hallii*, and *Alistipes putredinis* were inversely linked with T2DM risk in the prediction model (*p* < 0.00005; Figure 4B). The LDA at the genus and species levels revealed that *Bacteroides*, *Facealibacterium*, *Blautia*, *Anaerobutyricum*, *Alistipes*, *Anaerostipes,* and *Roseburia* were higher in the healthy group, while *Collinsella*, *Bifidobacterium*, *Flavonifractor*, *Phascolarctobacterium*, *Streptococcus*, and *Flintibacter* were higher in the T2DM group in ET-L (*p* < 0.05; Supplementary Figure S4A,B).

### 3.5. In ET-P, the Prediction of Fecal Microbiota Specific for T2DM by the Machine Learning Approach

In ET-P, 414 fecal FASTA/Q files of 335 healthy and 79 T2DM adults were analyzed to make a prediction model for T2DM risk. The AUC and 95% CIs for the prediction models generated by the three programs are presented in Figure 4C. The AUC of the ROC in the prediction model with 25 species was 0.948, 0.966, and 0.924 in the XGBoost, random forest, and linear regression models, respectively (*p* < 0.0001; Table 2). The results suggested that the prediction model of fecal microbes to explain T2DM risk was a good fit. The accuracy, sensitivity, specificity, and precision of the prediction model were about 0.84, 0.70, 0.93, and 0.74, and these values indicated that the model was acceptable. *Megasphaera elsdenii*, *Escherichia fergusonii*, *Desulfovibrio simplex*, *Oscillibacter valericigenes*, *Prevotella copri*, and *Megasphaera massiliensis* were higher, and *Bacteroides koreensis*, *Faecalibacterium prausnitzii*, *Clostridium polysaccharolyticum*, *Phocaeicola plebeius*, *Veillonella tobesuensis*, and *Intestinimonas butyriciproducens* were lower in the T2DM group than in the healthy group (*p* < 0.00005; Figure 4D). The LDA at the genus and species levels revealed similar to XGboost results ((*p* < 0.05; Supplementary Figure S4C,D).

### 3.6. Fecal Bacteria Interaction Network

**Figure 5:**
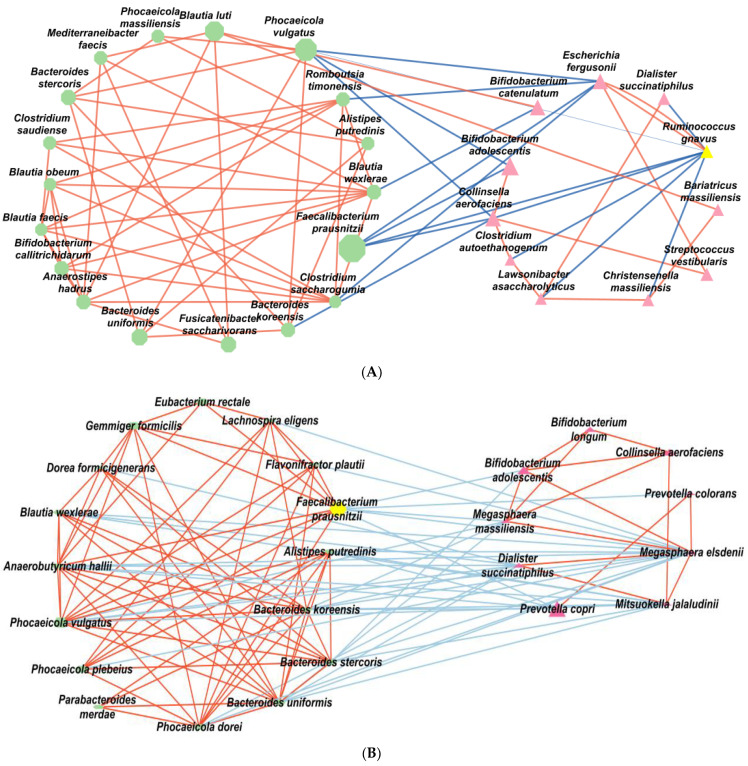
Network of primary bacteria in the T2DM and healthy groups according to each enterotype; (**A**) ET-L; (**B**) ET-P. Red and blue lines indicate positive and negative correlations with Pearson correlation coefficients > 0.1, respectively. ET-L: Enterotype Lachnospiraceae; ET-P: Enterotype Prevotellaceae.

Bacteria in the healthy and T2DM groups were selected from the results of SHAP. Bacteria in the same genus are positively or negatively associated with T2DM risk and interact with each other. As they co-occur in the gut, a colocalized network analysis was carried out. In ET-L, bacteria in the healthy and T2DM groups were separated, as shown in Figure 5A. The predominant bacteria within the healthy and the T2DM groups positively interacted with each other (*p* < 0.0001). However, the above bacteria in the healthy group were negatively correlated with those in T2DM. In the network analysis, *Phocaeicolar vulgatus*, *Romburia timonensis*, *Blautia wexlerae*, *Faecalibacterium prausnitzii*, and *Clostridium saccharogumia*, which were rich in the healthy group, were negatively correlated with *Escherichia fergusonii*, *Bifidobacterium catenulatum*, *Bifidobacterium adolescentis*, *Collinsella aerofaciens*, *Dialister succinatiphilus*, and *Ruminococcus gnavus* (*p* < 0.00005; Figure 5A). 

In ET-P, Faecalibacterium prausnitzii, Phocaeicolar vulgatus, Phocaeicola plebeius, Phocaeicola dorei, Bacteroides uniformis, Bacteroides koreensis, Blautia wexlerae, Germmiger formicillus, and Eubacterium rectale positively interacted in the healthy group. Their network possibly prevented the development of T2DM (*p* < 0.0001; Figure 5B). In the T2DM group, *Prevotella copri*, *Prevotella colorans*, *Bifidobacterium longum*, *Bifidobacterium adolescentis*, *Collinsella aerofaciens*, *Megashaera elsdenii*, and *Megashaera massiliensis* were positively correlated with each other (*p* < 0.00005). They negatively interacted with the bacteria linked to the healthy group (Figure 5B). Some bacteria in the healthy and T2DM groups existed in both ET-P and ET-L.

### 3.7. Metagenome Function

**Figure 6:**
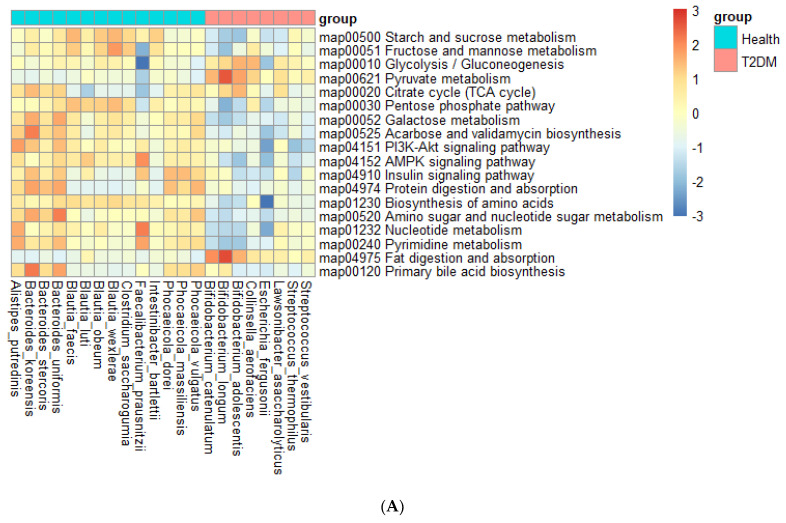

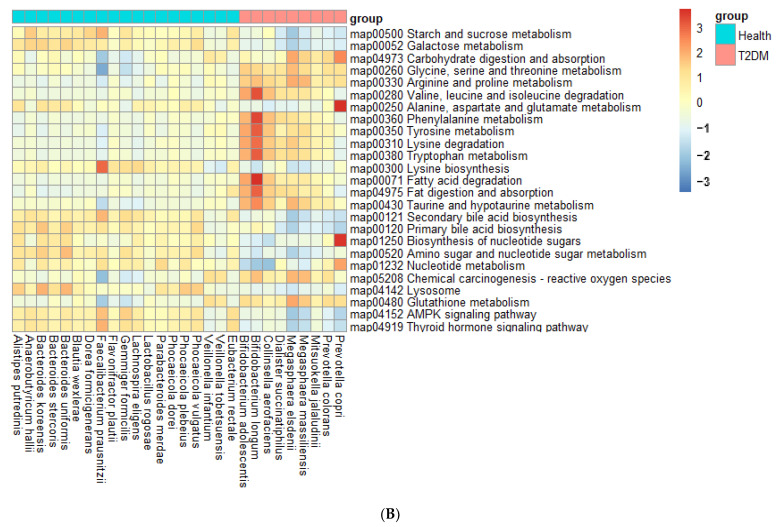
Metagenome functions of the primary bacteria according to each enterotype; (**A**) ET-L; (**B**) ET-P. ET-L: Enterotype Lachnospiraceae; ET-P: Enterotype Prevotellaceae.

In ET-L, gut bacteria in the T2DM group were positively correlated with glycolysis and gluconeogenesis, pyruvate metabolism, the tricarboxylic acid (TCA) cycle, and fat digestion and absorption, while carbohydrate metabolism, including starch, fructose, and mannose as well as galactose metabolism, pentose phosphate metabolism, amino acid metabolism, nucleotide metabolism, insulin signaling, and adenosine 5′ monophosphate-activated protein kinase (AMPK) signaling were negatively correlated (*p* < 0.00005; Figure 6A). Interestingly, acarbose biosynthesis was positively correlated with the bacteria in the T2DM group. 

In ET-P, fecal bacteria in the T2DM group were negatively correlated with starch, mannose, sucrose, and fructose metabolism. However, higher glycolysis, gluconeogenesis, and pyruvate and fat digestion and absorption were observed in the T2DM than in the healthy group in the participants with ET-P (*p* < 0.00005; Figure 6B). Unlike in ET-L, amino acid metabolism was positively correlated with fecal bacteria predominant in the T2DM group in the participants with ET-P. The PI3-Akt signaling, AMPK signaling, and insulin signaling pathways, amino sugar and nucleotide sugar metabolism, and primary bile acid biosynthesis were negatively correlated with the fecal bacteria predominant in the T2DM group (*p* < 0.00005). The metagenome functions in ET-L and ET-P suggested that glucose availability was higher in the gut of T2DM patients with ET-L and ET-P. The gut bacteria used glucose, while the utilization of other carbohydrates and protein were reduced (Figure 6B). As a result, gut microbiota in the T2DM group attenuated insulin and AMPK signaling to exacerbate the T2DM state of the host. 

## 4. Discussion

Accumulating evidence in recent years has demonstrated that the gut microbiome and its metabolites play a crucial role in the onset and development of many metabolic diseases, including T2DM. The composition of gut microbes drastically impacts the development and function of the PNS. The afferent, efferent, and enteric nervous systems are interconnected to respond to gut microbe signaling and together control various functions, including digestion and metabolism. Dramatic changes in diet and lifestyle, leading to obesity and T2DM, are linked to a disconnect between the altered gut microbiota and the PNS [26,27]. It is well-known that the autonomic nervous system modulates insulin secretion and plays an essential role in insulin resistance in T2DM [28]. The current data suggest that modulating the gut microbiome is a potential therapeutic approach to T2DM prevention and management. However, the currently available data on the role of the gut bacteria involved in the pathogenesis of T2DM are controversial [9,10,18,29]. The present study demonstrated that T2DM was associated with gut dysbiosis involving an increase in opportunistic pathogens as well as beneficial bacteria such as *Bifidobacterium*, which may be related to the autonomous nervous system, especially to activate vagal pathways independent of enterotypes. Fecal microbiota transplantation leads to the increased and sustained presence of favorable gut microbiota in patients with T2DM, which can be considered a therapeutic target for T2DM. 

A relative abundance of Firmicutes and Proteobacteria, an increase in F/B ratios, and a decrease in Bacteroidetes were observed in T2DM compared to the healthy group [18]. However, the results have not separated the participants based on enterotypes. Since the participants with different enterotypes have demonstrated different bacterial communities, fecal bacterial co-occurrence needs to be studied according to the enterotypes. In the present study, the participants with and without T2DM were separated into ET-L and ET-P and showed totally different bacterial compositions. Overall, F/B was higher in the T2DM group in both enterotypes [9,18]. However, the relative abundance of Firmicutes was lower in the T2DM group of ET-L and higher in the T2DM group of ET-P. Proteobacteria was higher in the T2DM group only in ET-P, and Actinobacteria was higher in both enterotypes. Some bacteria showed higher relative abundance in the T2DM group compared to the healthy group in both ET-L and ET-P, but other bacteria related to T2DM were predominant in one enterotype. Bacteria in different enterotypes have different bacterial networks to increase beneficial bacteria and decrease opportunistic pathogens. Therefore, the bacterial composition is closely but discretely related to T2DM risk according to enterotypes, and their modulation can be a therapeutic target for T2DM. These results can be applied to design personalized nutrition for Asians.

Fecal bacteria are known to be mainly divided into ET-B, ET-R, and ET-P in humans. In the present study, the fecal bacteria were stratified into two groups, ET-L and ET-P, using PCA analysis. The fecal bacteria composition of ET-L was between those of ET-B and ET-R. However, ET-B and ET-R were not separated by different distance matrices, including weighted/unweighted UniFrac, Euclidean, Bray–Curtis, and Jensen–Shannon divergence [30]. Dietary intake, lifestyles, antibiotic administration, host genetics involved in the vagus nerve, bile acid metabolism, and digestion ability influence enterotypes [11,14]. People on a long-term intake high in animal protein and fat are susceptible to becoming ET-B; those consuming a lower energy intake and a high-carbohydrate diet, especially simple sugars, are likely to belong to ET-P; and those on various food intakes are likely to be in ET-R. ET-B is mainly related to bile acid metabolism [6]. However, short-term dietary interventions do not shift one enterotype to another [31] since the gut microbiota are colocalized and have a network to support the survival of the bacteria in each enterotype. Furthermore, the host’s metabolism, modulated by genetics and vagus nerve activation, influences the gut microbiota. Enterotypes are not discrete; they are continuous, with a distinct overlap of bacteria [31]. The bacterial community in the enterotypes with overlap can be switched under specific circumstances, such as changing diets. In a randomized controlled trial (RCT) with Korean-style and westernized-style diets, the participants were observed to be ET-B and ET-P. Only 10–15% of them switched enterotype, mainly those with overlapping bacteria compositions despite different enterotypes [32]. Therefore, the enterotype itself does not act as a biomarker for a specific disease, but the bacteria affecting the diseases need to be isolated in each enterotype.

The vagus nerve activates the cholinergic nervous system to promote an anti-inflammatory pathway and decreases intestinal permeability to modulate gut bacterial composition [27,33]. Gut microbiota in children and elderly persons are quite different from those in adults [34], which is related to the maturation and degeneration of the vagus nerve. Infants have high levels of *Bifidobacterium* as beneficial bacteria and Clostridiaceae and Enterobacteriaceae as harmful bacteria, possibly linked to an immature vagus nerve system [34]. Scopolamine administration suppresses the parasympathetic nervous system in a rat model and induces gut dysbiosis by decreasing *Bacteroides* and *Faecalibacterium prausnitzii* and increasing *Ruminococcus* and *Clostridium* in the feces [27]. In the present study, the fecal bacteria of adults with and without T2DM were included as samples. In ET-L, the relative abundance of *Phocaicola*, *Bacteroides*, *Blautia*, *Faecallibacterium*, and *Roseburia* was higher in the healthy group than the T2DM group, but that of *Bifidobacterium, Streptococcus*, *Collinsella*, and *Dialister* was higher in the T2DM group than the healthy group. Although the results cannot be explained using a cause-and-effect relationship, these bacteria are related to T2DM, and they indicate that the vagus nervous system could be suppressed in T2DM.

Previous studies have demonstrated that *Bifidobacterium*, *Bacteroides*, *Faecalibacterium*, *Akkermansia*, and *Roseburia* are negatively associated, while *Ruminococcus*, *Fusobacterium*, and *Blautia* are positively associated with T2DM [9,18]. *Bifidobacterium* such as *Bifidobacterium adolescentis*, *Bifidobacterium bifidum*, *Bifidobacterium pseudocatenulatum*, *Bifidobacterium longum*, and *Bifidobacterium dentium* have benefits in T2DM and can improve host glucose tolerance and glucose-induced insulin secretion as well as reduce inflammation [35]. Treatment with the oral hypoglycemic agent metformin or gastric bypass surgery increases Bifidobacterium species such as *Bifidobacterium adolescentis* [29,36]. However, Sasaki et al. have demonstrated that *Lactobacillus* and *Bifidobacterium* are significantly more abundant in T2DM patients than in healthy adults [37]. In the present study, the relative abundance of *Bifidobacterium* such as *Bifidobacterium adolescent* and *Bifidobacterium longum* was higher in the T2DM group than in the healthy group; they had a positive interaction with some bacteria in the healthy group and no positive interaction with bacteria linked to immunity in the T2DM group. Previous studies have demonstrated that decreased sympathetic and vagal nerve functional states are exhibited in Asians with T2DM, independent of their HbA1c levels [38]. Therefore, the increase in *Bifidobacterium* in T2DM may be related to a disturbed vagus nervous system and the higher availability of glucose and oligosaccharides in the large intestines and colons in Asians with T2DM. When the vagus nervous system of the parasympathetic nervous system is suppressed, an increase in *Bifidobacterium* might prevent the exacerbation of T2DM symptoms and the induction of the related complications compared to an increase in Proteobacteria such as *Escherichia*.

The gut bacteria interact with the host, acting as innocuous commensals, opportunistic pathogens, or probiotics to modulate host immunity and metabolism. *Bifidobacterium* and *Lactobacillus* are well-known probiotics that decrease proinflammatory cytokines, short-chain fatty acids (SCFA), and organic acids to alter the host’s metabolism [39]. However, specific bacterial growth associated with *Bifidobacterium* is positively associated with commensal bacteria such as *Collinsella aerofaciens*, *Megasphaera massiliensis*, *Megasphaera elsdenii*, and *Dialister succinatiphilus*. *Megasphaera* belongs to the family Veillonellaceae, and *Dialister* is the nearest relative of *Megashaera*. Their species positively interacted to increase in abundance in T2DM and negatively interacted with *Faccalibacterium prausnitzii*, *Bacteroides koreensis*, *Bacteroides uniformis*, *Bacteroides stereopsis*, and *Phocaeicola vulgatus*, primarily in ET-P. In ET-L, *Ruminococcus gnavus*, *Escherichia fergusonii*, *Streptococcus vestibularis*, *Collinsella aerofaciens*, *Clostridium autoethanogenum*, and *Lawsonbacter asaccharolyticus* made a network. However, *Ruminococcus gnavus* had negative interactions with *Clostridium autoethanogenum*, *Collinsella aerofaciens*, and *Lawsonbacter asaccharolyticus* in T2DM. *Streptococcus vestibularis* potentially promoted harmful bacteria that increased T2DM risk, and *Faecalibacterium prausnitzii, Phocaeicola vulgatus*, and *Clostridium saccharogumia* protected against the increment in bacteria linked to T2DM in persons with ET-L. These results suggest that *Faecalibacterium prausnitzii* and *Phocaeicola vulgatus* potentially play a critical role in protecting against gut dysbiosis by decreasing T2DM-related bacteria.

The present study had some strengths. It included a relatively high sample size of T2DM (*n* = 551) and healthy (*n* = 3378) groups. They were stratified into two groups according to bacterial compositions, ET-L and ET-P. The study results provided some clues on ways to suppress the vagus nervous system in T2DM patients independent of enterotypes. The primary bacteria in the T2DM group were *Ruminococcus gnavus* in ET-L and *Prevotella copri* in ET-P, which interacted with other species. The primary bacteria for T2DM can be therapeutic markers, and the primary bacteria can be eliminated by promoting them to have networks in ET-L and ET-P. This study had some limitations. The fecal FASTA/Q data in the adults were collected from the NCBI database. However, the demographic characteristics of the participants were not provided, and the data were analyzed by adjusting for demographic information. Information on the drugs used to treat T2DM and nutrient and antibiotic intake, which influence gut microbiota composition, was limited and could not be controlled to identify potential confounders. The data were collected in case-control studies, and the results could not be applied to evaluate cause and effect.

## 5. Conclusions

Asians with T2DM have different fecal bacterial communities with their co-abundance networks and metagenome functions compared to healthy individuals. The results suggest that the bacterial differences influence the host’s glucose metabolism, which could be related to the disturbance of the vagus nervous system in both the ET-L and ET-P enterotypes. Although the F/L ratio was higher in T2DM than in healthy adults in both enterotypes, Proteobacteria in ET-P and Actinobacteria in ET-L were much higher in T2DM than the healthy adults. In ET-L, the increment in *Escherichia fergusonii*, *Collinsella aerofaciens*, *Streptococcus vestibularis*, and *Streptococcus periodonticum* increased T2DM risk, but increased *Bifidobacterium* could lower the T2DM risk. In ET-P, *Escherichia fergusonii*, *Megasphaera elsdenii*, *Oscillibacter valericigenes*, *Prevotella copri*, and *Megasphaera massiliensis* were higher, indicating increased Enterobacteriaceae without increasing *Bifidobacterium* in T2DM. Furthermore, the increment in *Bifidobacterium* and Enterobacteriaceae suggests the potential suppression of the vagus nervous system, thereby influencing T2DM risk. An increase in *Bifidobacterium* might benefit T2DM symptoms, but an increase in Enterobacteriaceae could worsen T2DM. The bacterial differences also influence insulin and AMPK signaling, contributing to the alterations of the host’s glucose metabolism. In both enterotypes, *Faecalibacterium prausnitzii* protected against the growth of harmful bacteria in T2DM. Therefore, the modulation of the PNS can alter gut microbiota that may be involved in increasing T2DM risk. Future research is needed to identify therapeutic agents to decrease the harmful bacteria and increase beneficial bacteria in T2DM, which may prevent and alleviate T2DM by producing gut eubiosis in Asians. Moreover, the direct relationship between the impairment of the vagus nervous system and intestinal permeability contributing to T2DM development and progression should be determined in animal and human studies. Since the T2DM etiology of Asians and Caucasians is known to be different, the fecal bacteria composition and metagenome functions need to be determined in Caucasians and compared.

## Figures and Tables

**Table 1:** Characteristics of human fecal samples collected from the National Center for Biotechnology Information (NCBI) website.

Country	Enterotype	No. Projects	No. T2DM	No. Healthy	Total Sample	Age	BMI	No. Male	No. Female	No. Gender
China	ET-L	22	19	2251	2270	43.9	23.3	78	52	2140
	ET-P	16	1	256	257	44.6	22.8	12	3	242
India	ET-L	6	87	79	166	-	-	-	-	166
	ET-P	6	61	44	105	-	-	-	-	105
Japan	ET-L	4	366	708	1074	44.3	-	156	213	705
	ET-P	4	17	33	50	45	-	2	0	48
Thailand	ET-L	1	0	5	5	-	-	5	0	0
	ET-P	1	0	2	2	-	-	2	0	0

ET-L: Enterotype Lachnospiraceae; ET-P: Enterotype Prevotellaceae; T2DM: Type 2 diabetes mellitus; BMI: Body mass index.

**Table 2:** Accuracy, sensitivity, specificity, and precision in the prediction model generated with a machine learning approach, according to enterotypes.

**ET-L**	**XGBoost**	**Random Forest**	**Linear Regression**
10-fold crossover	0.913 (0.913–0.914)	0.912 (0.912–0.913)	0.911 (0.910–0.911)
AUC	0.948 (0.947–0.948)	0.966 (0.966–0.967)	0.924 (0.923–0.924)
Accuracy	0.900 (0.900–0.901)	0.896 (0.895–0.897)	0.890 (0.890–0.891)
Sensitivity	0.753 (0.750–0.756)	0.721 (0.719–0.724)	0.780 (0.777–0.783)
Specificity	0.969 (0.968–0.969)	0.990 (0.989–0.990)	0.952 (0.951–0.952)
Precision	0.878 (0.874–0.880)	0.940 (0.939–0.941)	0.802 (0.700–0.705)
**ET-P**	**XGBoost**	**Random Forest**	**Linear Regression**
10-fold crossover	0.864 (0.864–0.865)	0.851 (0.850–0.852)	0.842 (0.841–0.842)
AUC	0.901 (0.899–0.904)	0.949 (0.948–0.951)	0.913 (0.911–0.915)
Accuracy	0.843 (0.840–0.845)	0.865 (0.863–0.867)	0.887 (0.885–0.889)
Sensitivity	0.703 (0.695–0.710)	0.687 (0.681–0.695)	0.737 (0.728–0.747)
Specificity	0.929 (0.927–0.931)	0.986 (0.985–0.987)	0.922 (0.921–0.924)
Precision	0.742 (0.733–0.750)	0.876 (0.869–0.884)	0.740 (0.731–0.749)

ET-L: Enterotype Lachnospiraceae; ET-P: Enterotype Prevotellaceae; AUC: Area under the curve.

## Data Availability

The authors confirm that the data supporting the findings of this study are available within the article and its Supplementary Materials. The FASTA/Q files for human studies were downloaded from the NCBI, and the data for the animal studies are available upon request from the corresponding author.

## Supplementary Materials

The following supporting information can be downloaded at: https://www.mdpi.com/article/10.3390/biomedicines10112998/s1, Figure S1. Chao1 and Shannon indices representing α-diversity according to enterotypes. (A) Chao1 index in ET-L; (B) Shannon index in ET-L; (C) Chao1 index in ET-P; (D) Shannon index in ET-P; ET-L: Enterotype Lachnospiraceae; ET-P: Enterotype Prevotellaceae; Figure S2. B-diversity according to enterotypes; (A) B-diversity in ET-L; (B) B-diversity in ET-P, ET-L: Enterotype Lachnospiraceae; ET-P: Enterotype Prevotellaceae; Figure S3. Relative abundance of bacteria at the phylum level according to enterotypes; (A) ET-L; (B) ET-P; (C) The ratio of Firmicutes and Bacteroidetes (F/L), * Significantly different between T2DM and healthy groups at *p* < 0.05, ** at *p* < 0.01, and *** at *p* < 0.001. ET-L: Enterotype Lachnospiraceae; ET-P: Enterotype Prevotellaceae; Figure S4. Primary bacteria at the genus and species levels between type 2 diabetes and healthy groups by LDA according to the enterotypes; (A) Linear discriminant analysis (LDA) scores of bacteria at the genus level in ET-L; (B) Linear discriminant analysis (LDA) scores of bacteria at the species level in ET-L; (C) Linear discriminant analysis (LDA) scores of bacteria at the genus level in ET-P; (D) Linear discriminant analysis (LDA) scores of bacteria at the species level in ET-P, ET-L: Enterotype Lachnospiraceae; ET-P: Enterotype Prevotellaceae.
