# Supplementary material for: Fecal Bacterial Community and Metagenome Function in Asians with Type 2 Diabetes, According to Enterotypes
Source: Biomedicines. 2022 Nov 21;10(11):2998. doi: 10.3390/biomedicines10112998 (PMC9687834; doi:10.3390/biomedicines10112998)
Supplement: Supplementary file 1 [file biomedicines-10-02998-s001.zip › biomedicines-2008843-supplementary.pdf]

**Supplementary figures**
**Supplementary Figures S1–S4**

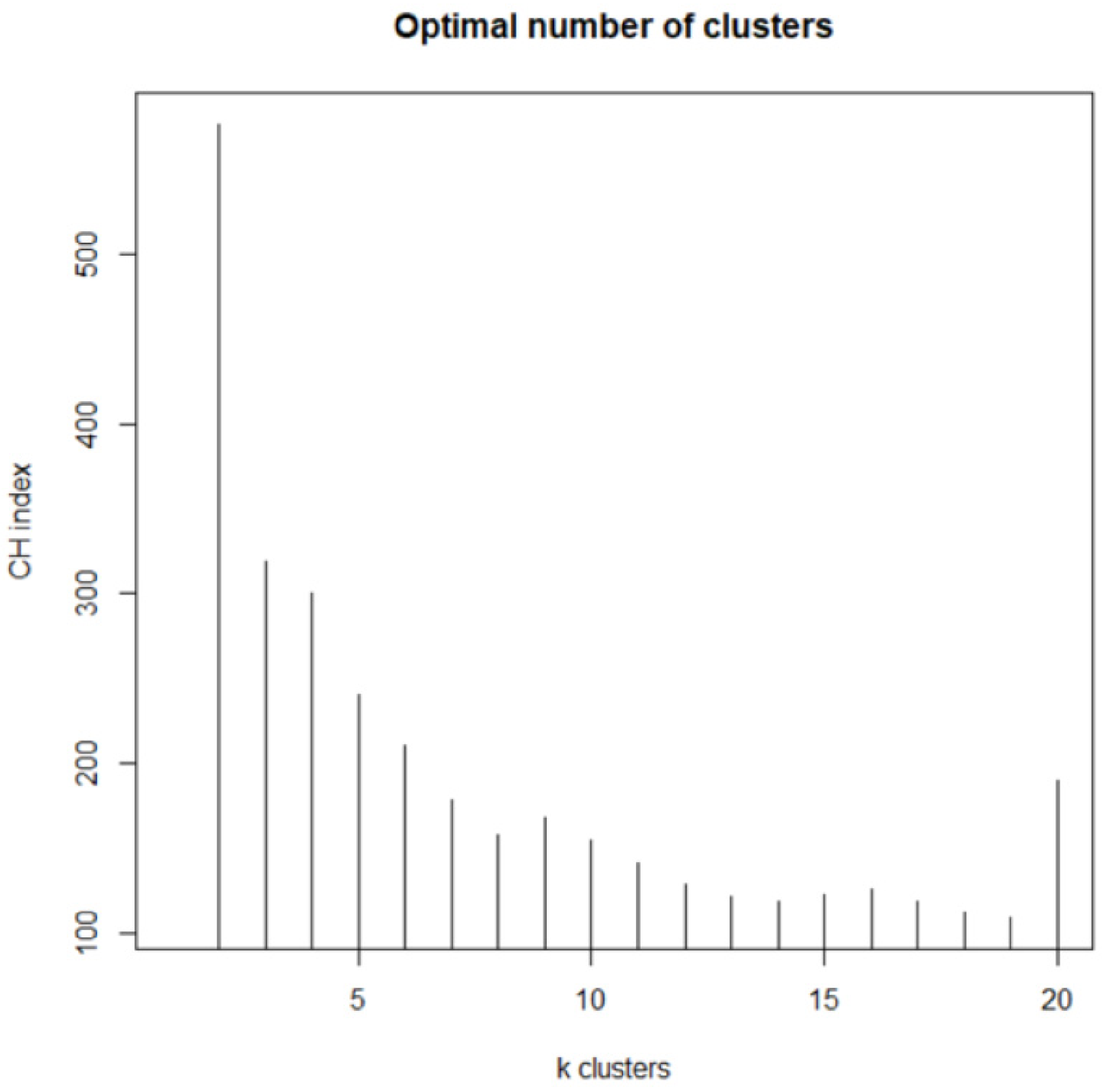

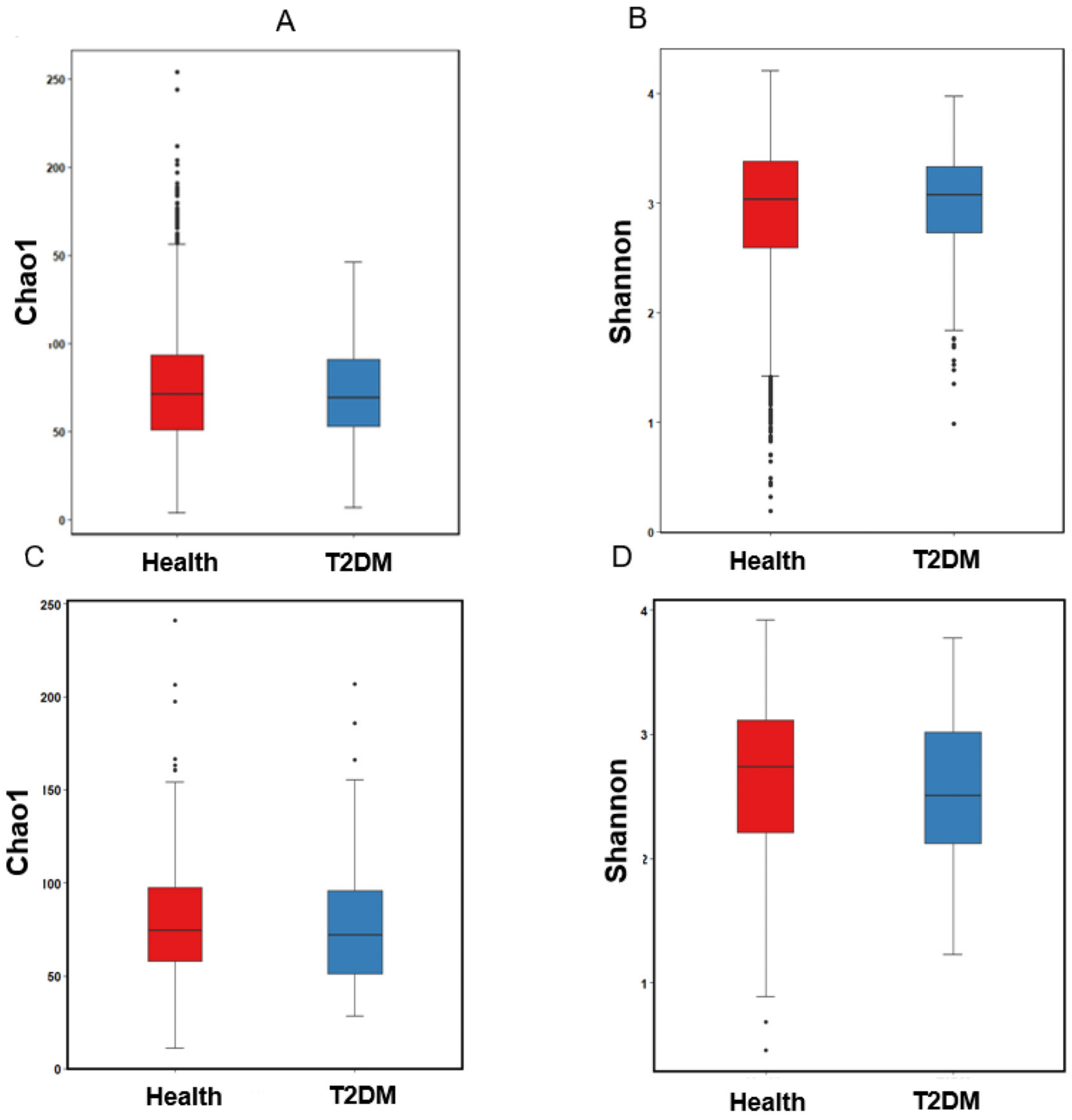


**Figure S1.** Chao1 and Shannon indices representing $\alpha$-diversity according to enterotypes. (A).Chao1 index in ET-L. (B). Shannon index in ET-L. (C). Chao1 index in ET-P. (D). Shannon index in ET-P

ET-L: Enterotype- Lachnospiraceae; ET-P: Enterotype-Prevotellaceae

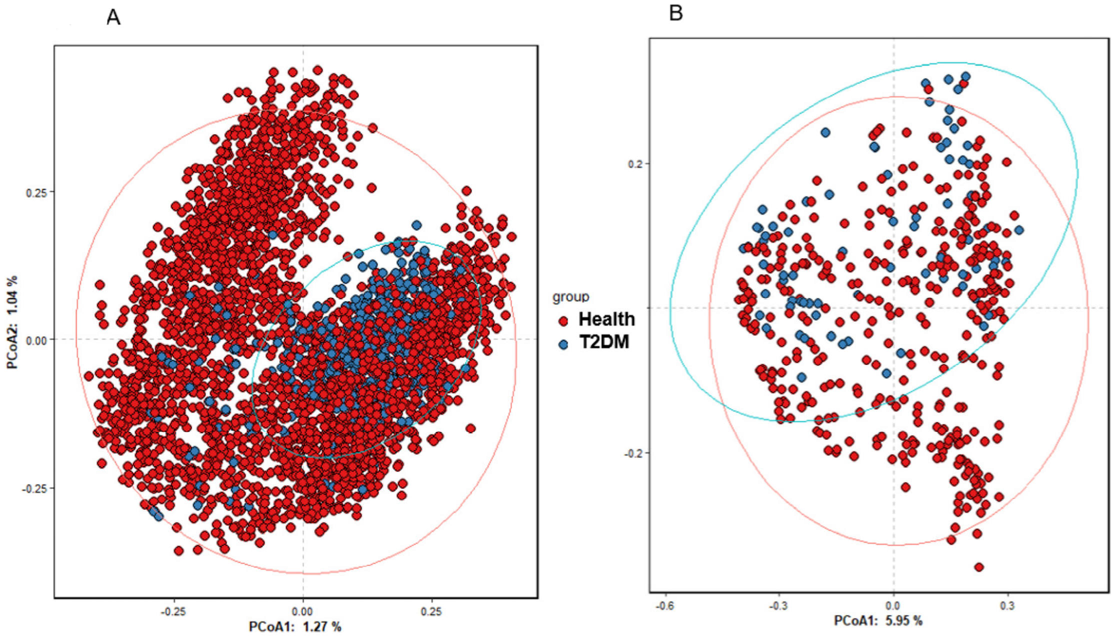


**Figure S2.** B-diversity according to enterotypes. (A). B-diversity in ET-L. (B). B-diversity in ET-P.

ET-L: Enterotype-Lachnospiraceae; ET-P: Enterotype-Prevotellaceae

A

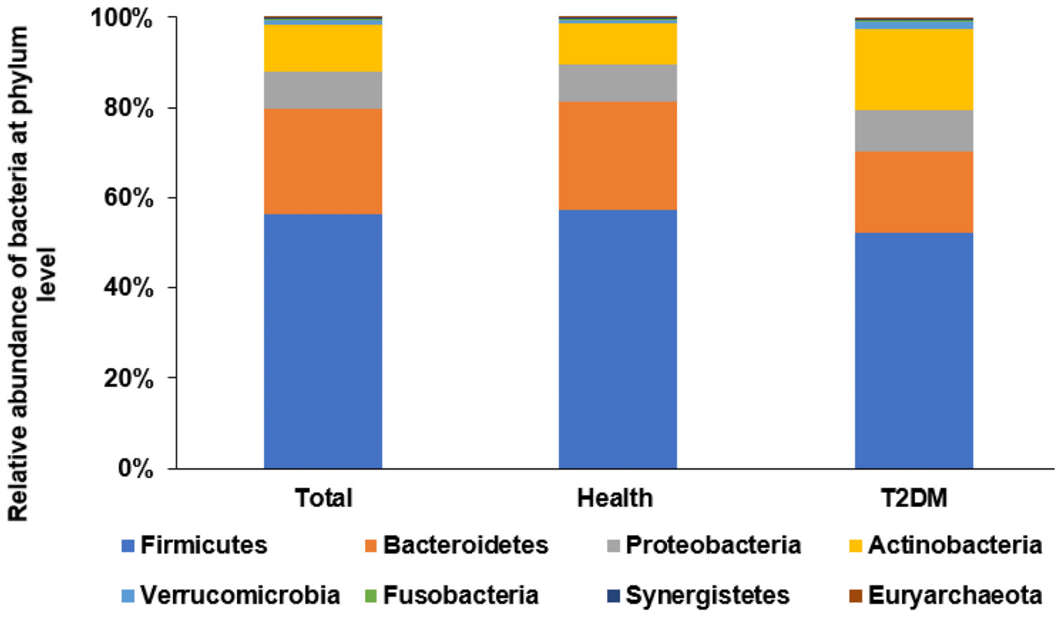
Relative abundance of bacteria at phylum level
100%
80%
60%
40%
20%
0%
Total
Health
T2DM
Firmicutes
Bacteroidetes
Proteobacteria
Actinobacteria
Verrucomicrobia
Fusobacteria
Synergistetes
Euryarchaeota

B

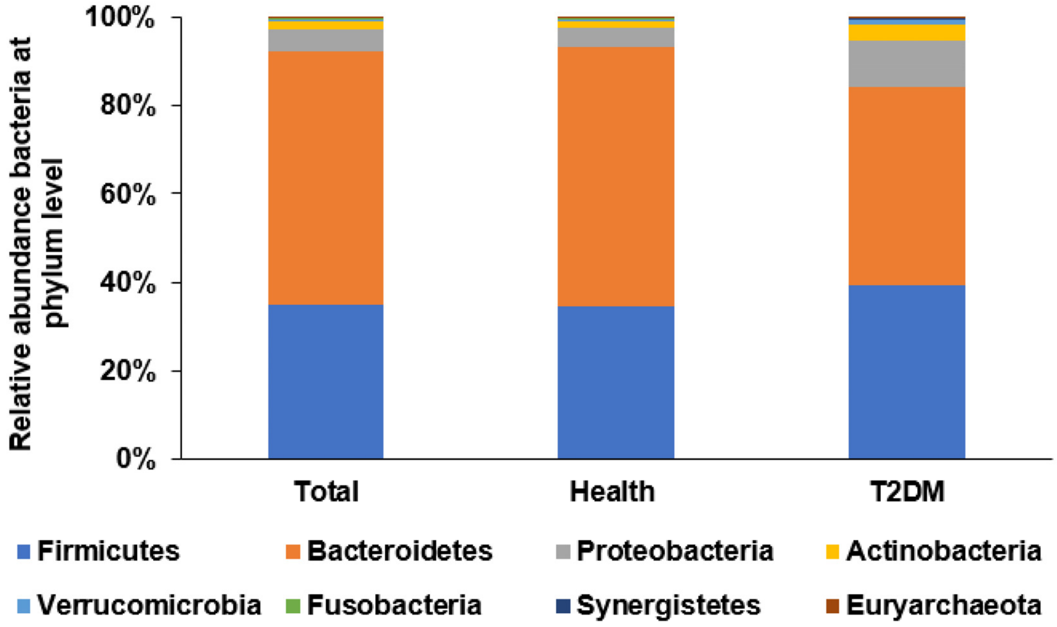
Relative abundance bacteria at phylum level
100%
80%
60%
40%
20%
0%
Total
Health
T2DM
Firmicutes
Bacteroidetes
Proteobacteria
Actinobacteria
Verrucomicrobia
Fusobacteria
Synergistetes
Euryarchaeota

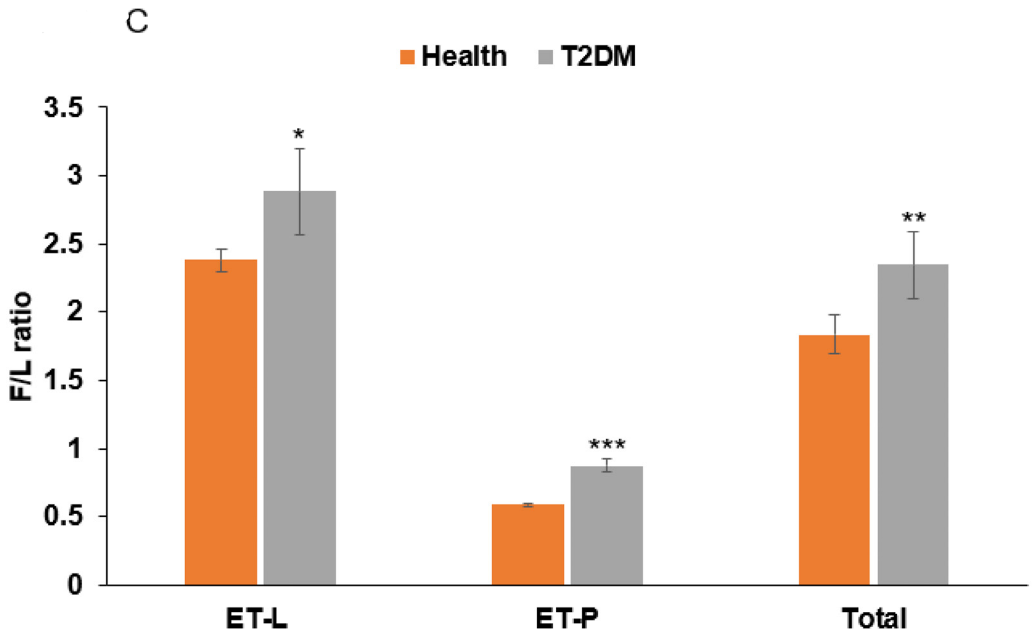


**Figure S3.** Relative abundance of bacteria at the Phylum level according to enterotypes. (A). ET-L. (B). ET-P. (C). The ratio of Firmicutes and Bacteroidetes (F/L). * Significantly different between T2DMand Healthy groups at $p < 0.05$, ** $p < 0.01$, and *** $p < 0.001$. ET-L: Enterotype-Lachnospiraceae; ET-P: Enterotype-Prevotellaceae

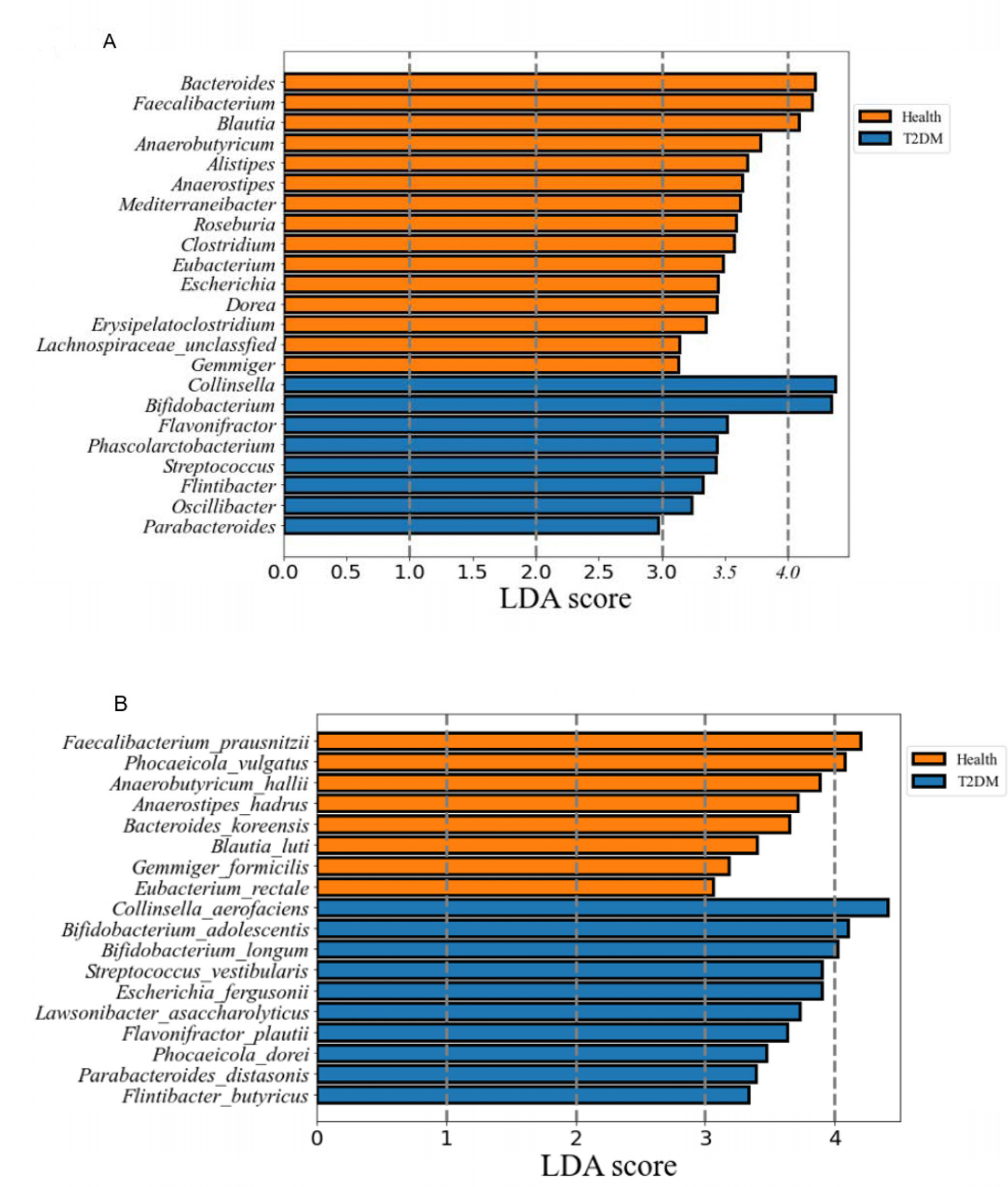
A
Bacteroides
Faecalibacterium
Blautia
Anaerobutyricum
Alistipes
Anaerostipes
Mediterraneibacter
Roseburia
Clostridium
Eubacterium
Escherichia
Dorea
Erysipelatoclostridium
Lachnospiraceae_unclassfied
Gemmiger
Collinsella
Bifidobacterium
Flavonifractor
Phascolarctobacterium
Streptococcus
Flintibacter
Oscillibacter
Parabacteroides
Health
T2DM
0.0 0.5 1.0 1.5 2.0 2.5 3.0 3.5 4.0
LDA score
B
Faecalibacterium_prausnitzii
Phocaeicola_vulgatus
Anaerobutyricum_hallii
Anaerostipes_hadrus
Bacteroides_koreensis
Blautia_luti
Gemmiger_formicilis
Eubacterium_rectale
Collinsella_aerofaciens
Bifidobacterium_adolescentis
Bifidobacterium_longum
Streptococcus_vestibularis
Escherichia_fergusonii
Lawsonibacter_asaccharolyticus
Flavonifractor_plautii
Phocaeicola_dorei
Parabacteroides_distasonis
Flintibacter_butyricus
Health
T2DM
0 1 2 3 4
LDA score

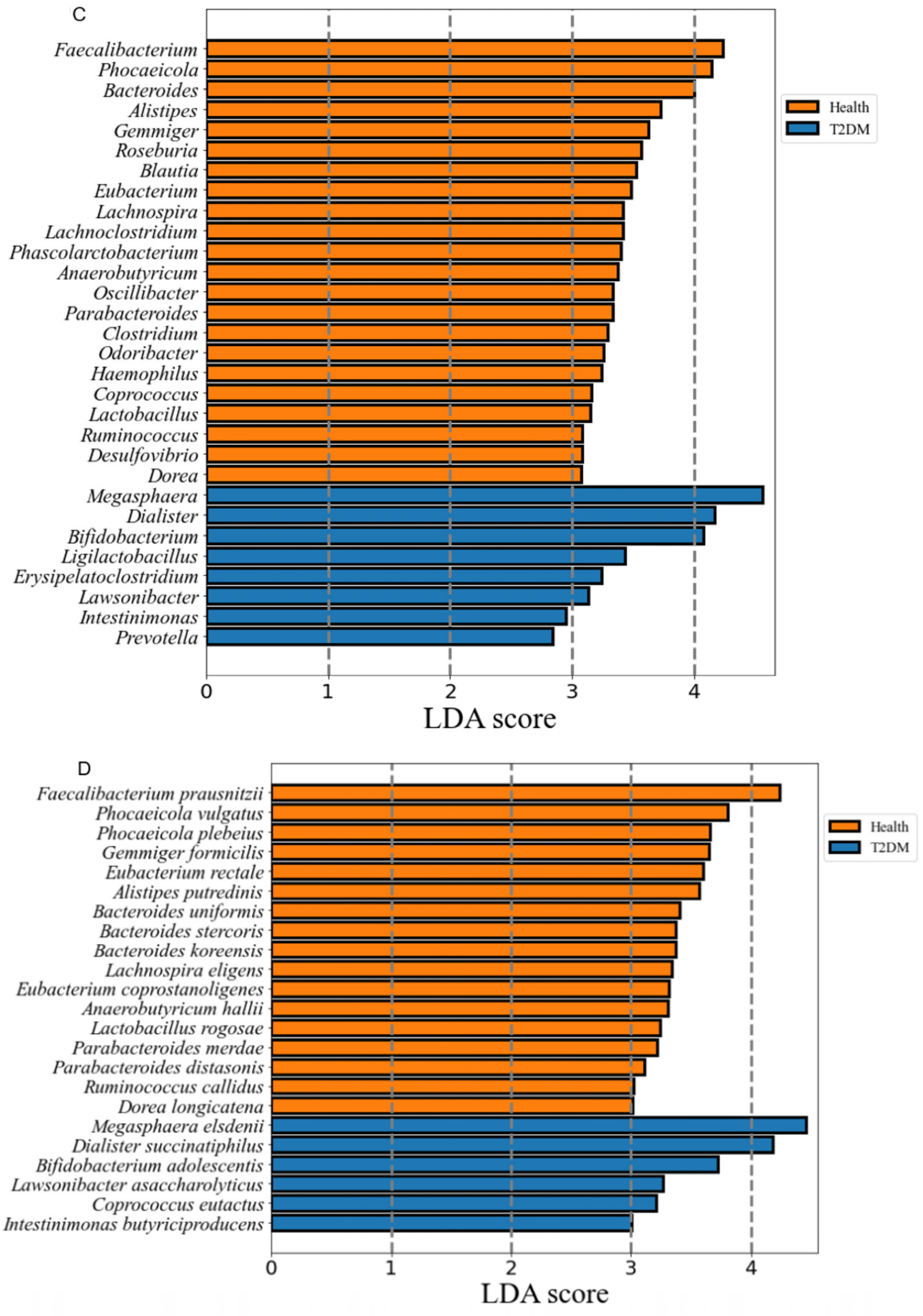


**Figure S4.** Primary bacteria at the genus and species between type 2 diabetes and Healthy groups by LDA analysis according to the enterotypes. (A). Linear discriminant analysis (LDA) scores of bacteria at the genus level in ET-L. (B). Linear discriminant analysis (LDA) scores of bacteria at the species level in ET-L. (C). Linear discriminant analysis (LDA) scores of bacteria at the genus level in ET-P. (D). Linear discriminant analysis (LDA) scores of bacteria at the species level in ET-P.

ET-L: Enterotype-Lachnospiraceae; ET-P: Enterotype-Prevotellaceae
